# A Pilot, Open-Label Study to Evaluate the Efficacy of Intra-Articular Administration of a Caninized TNF Receptor Fc Fusion Protein as a Treatment for Osteoarthritis-Associated Joint Pain

**DOI:** 10.3389/fvets.2022.836709

**Published:** 2022-06-02

**Authors:** Aoi Nakanishi, B. Duncan X. Lascelles, Julie Allen, Beth Case, David Gearing, Masataka Enomoto

**Affiliations:** ^1^Department of Animal Science, College of Agriculture and Life Sciences, North Carolina State University, Raleigh, NC, United States; ^2^Translational Research in Pain (TRiP) Program, Department of Clinical Sciences, College of Veterinary Medicine, North Carolina State University, Raleigh, NC, United States; ^3^Department of Clinical Sciences, Comparative Pain Research and Education Center, North Carolina State University, Raleigh, NC, United States; ^4^Thurston Arthritis Center, UNC School of Medicine, Chapel Hill, NC, United States; ^5^Center for Translational Pain Research, Department of Anesthesiology, Duke University, Durham, NC, United States; ^6^Department of Population Health and Pathobiology, North Carolina State University, Raleigh, NC, United States; ^7^The Centre for Innate Immunity and Infectious Diseases, Hudson Institute, Melbourne, VIC, Australia

**Keywords:** osteoarthritis, joint pain, TNF-α, force plate, limb use asymmetry test

## Abstract

Tumor necrosis factor-α (TNF-α) is a potential target for osteoarthritis (OA) treatment. In several recent clinical studies in human OA, anti-TNF-α therapy showed promising results; however, these were open-label and based on patient-reported outcome measures. In this study, we developed a caninized TNF-α receptor-Fc (caTNFR-Fc) fusion protein and conducted a non-randomized, open-label, pilot study in dogs with OA using objectively measured ground reaction forces and activity. The aims of the study were to assess the efficacy of the intra-articular (IA) injection of the caTNFR-Fc fusion protein as a treatment for OA pain, and additionally to evaluate TNF concentrations in synovial fluid (SF) between joints with/without OA in dogs. Dogs (*n* = 12) with single-limb lameness due to single joint appendicular OA were recruited. All dogs received caTNFR-Fc fusion protein injection into the affected joint under sedation. Objective kinetic gait analysis using force plate was performed prior to (baseline), and at 14- and 28-days following treatment. Additionally, SF samples were collected from OA joints (*n* = 69) and non-OA joints (*n* = 79) in a different cohort of dogs and TNF-α were measured using enzyme-linked immunosorbent assay. No significant treatment effects on the limb use, activity, and the questionnaire were found. The concentration of TNF-α was significantly higher in OA joints than in healthy joints (*p* = 0.0019), but TNF-α was detected in only 10/69 OA samples. The IA injection of caTNFR-Fc fusion protein provided no benefit in terms of objective limb use and activity data in dogs with OA in this pilot study. Although the SF concentration of TNF-α was significantly higher in OA joints, few OA joints had measurable TNF-α. Collectively, the data indicate TNF-α may not be a good therapeutic target in canine OA.

## Introduction

Osteoarthritis (OA) is a highly prevalent disease in dogs, with an estimated minimum of 20%−30% of dogs clinically affected (1). However, recently, employing a screening checklist in general practices, investigators found ~37% of dogs presenting to these practices (in the US) had a diagnosis of presumed OA (2). OA can be a single or multiple joint disease(s), likely with multiple joint OA predominating although this has not been comprehensively studied. OA-related pain remains a challenging clinical entity to treat with limited approved or proven systemic therapeutics. Additionally, there are no proven or approved intra-articular drug therapies for dogs. Clinically, it is apparent that many dogs have one or two joints that are problematic with respect to OA-associated pain (e.g. uni- or bilateral elbow dysplasia cases), meaning there is a clinical therapeutic need for effective, safe, intra-articular therapies.

Tumor necrosis factor-α (TNF-α), a potent pro-inflammatory cytokine exerting pleiotropic effects *via* binding to TNF receptors (TNFRs) on various cell types, is known to play a role in the pathophysiological processes occurring in inflammatory degenerative joint diseases (3). TNF-α can be a major driver of nociception (4). With an increasing understanding of the role of TNF-α in joint pathology and pain, drugs targeting TNF-α have emerged as potentially useful therapeutic avenues for pain control, especially for the management of immune-mediated diseases (5). Etanercept, a human TNF receptor-Fc fusion protein, is a biopharmaceutical-approved medication for the treatment of autoimmune diseases by interfering with TNF-α. Etanercept has been shown to be effective in reducing signs and symptoms, such as pain and swelling, in patients with autoimmune diseases (6). Interestingly, a recent clinical study showed that a single injection of etanercept into knee joints with OA also significantly reduced the Western Ontario and McMaster Universities Osteoarthritis Index (WOMAC) score over the 4-week study period compared to intra-articular (IA) hyaluronic acid injection (7). However, this study was open-label, and the outcome measures were subjective. The placebo effect following IA injections is known to be significant (8), casting doubt on the efficacy seen in an open-label study. Additionally, there is debate over the pathological importance of TNF-α in OA-pain, in contrast to its clear role in rheumatoid arthritis pain (9).

In the present study, a fully caninized TNF receptor-Fc (caTNFR-Fc) fusion protein, similar to etanercept, was developed for potential use in dogs. We conducted the pilot study to evaluate the efficacy of IA injection of caTNFR-Fc fusion protein for the management of OA-pain in dogs using objective gait analysis and accelerometry data. Additionally, we analyzed synovial fluid (SF) and serum samples from dogs with and without OA to document the degree of upregulation of TNF-α in OA joints.

We hypothesized that IA injection of caTNFR-Fc fusion protein would decrease pain/inflammation associated with OA and therefore improve limb function and activity in dogs with OA-pain. The primary objectives of this pilot study were to explore the efficacy potential of the caTNFR-Fc fusion protein, and to investigate differences in the SF concentrations of TNF-a between the joints with and without OA. Secondary objectives were to evaluate the pharmacokinetics of caTNFR-Fc fusion protein following IA injection and to assess the possible adverse events (AEs) associated with caTNFR-Fc fusion protein in dogs.

## Materials and Methods

### Study Design

The study consisted of two separate parts. Part A was a non-randomized, open-label, pilot proof of principle study with all dogs receiving treatment. The Institutional Animal Care and Use Committee (IACUC) approved this study (IACUC # 15-163-O), and in all cases owners signed a written consent form following a detailed verbal explanation of the study protocol. In Part B, SF fluid and serum samples collected from dogs in Part A, and also from additional well-phenotyped dogs with and without OA, were analyzed for TNF-α concentrations. All samples were collected at North Carolina State University (NCSU) and conducted with the approval of IACUC (IACUC # 15-163-O, 16-144, 11-073-O, 13-010-B), and informed written consent from each owner.

### Study Population

#### Part A

Dogs ≥1-year old and ≥15kg with single limb lameness resulting from OA pain in a single joint were recruited for Part A. Recruitment was performed using NCSU websites, e-mails to the NCSU-College of Veterinary Medicine (CVM), and other university colleges. Flyers were distributed to the public at the NCSU-CVM Open House (April 2016). Promotion of the study was also accomplished using the NCSU-CVM social media sites (Twitter and Facebook posts). Recruitment began mid-January 2016 and continued through early May 2016. Twelve dogs were enrolled in this pilot study.

#### Part B

Serum and SF samples were collected from the client-owned dogs ≥15 kg presented to NCSU Veterinary Hospital for surgical intervention (cranial cruciate ligament rupture and fragmented medial coronoid process). Medical records, including patient history, operation record, medication history, and radiographic images were reviewed. SF samples were collected before opening the affected joint using a 22G needle during a surgery. Additionally, SF samples were gathered from the dogs ≥15kg that had been euthanized for reasons unrelated to this study. SF samples collected during Part A were also used. All dogs from which SF and serum samples were collected were evaluated carefully for the presence of OA and pain associated with OA.

### Inclusion Criteria for Part A

To be eligible for Part A of the study, dogs were required to have visible single limb lameness that was caused by OA-related pain in a single joint (carpus, elbow, shoulder, tarsus, or stifle), and required to have owner-detected signs of functional impairment (Liverpool Osteoarthritis in Dogs; LOAD scores of ≥10) (10, 11). This painful joint was the index joint. If there was pain and OA in more than 1 joint in the same limb, the index joint was required to be significantly worse than the others and was required to be the predominant reason for the lameness in the opinion of the investigators. If a predominantly affected joint could not be unequivocally identified as the cause of lameness of the limb, that dog was not included in the study. Additionally, dogs were required to have consistent measurable asymmetry in ground reaction forces (GRFs) between left and right forelimbs or left and right hindlimbs using a force plate (ideally, more than 5 percent BW difference). All examinations were performed by the same examiner (ME) throughout the study. All joints eliciting any degree of pain reaction from the dog during the examination were radiographed. Radiological features used to establish the presence of OA in appendicular joints were joint effusion, osteophytes, sclerosis, subluxation, subchondral bone erosions and cysts, and presence of intra-articular mineralization. Based on history, dogs were required to have had clinical OA (OA and associated pain/lameness) for 6 months and if OA in the stifle joint was due to a ruptured cruciate ligament, the rupture must have occurred at least 6 months prior to the date of inclusion and that stifle joint was required to be stabilized (based on palpation) by surrounding soft tissue or surgical repair (more than 3 months ago). The dogs were required to not be currently receiving any anti-inflammatory medications, or other analgesics (e.g., amantadine, gabapentin, tramadol). However, non-steroidal anti-inflammatory drugs (NSAIDs) were permitted if it was deemed there was currently significant pain associated with OA, and the dogs had been on NSAIDs for at least 3 weeks. Dogs were required to be either not receiving nutritional supplements or have been on them for 6 weeks or more before the start of the study. A 2-week withdrawal period was required prior to study entry for dogs discontinuing nutritional supplements, NSAIDs, or other analgesics. If dogs were considered to be mobility impaired but no OA was detected radiographically, or if they had OA but the impairment in mobility was not sufficient, they were not enrolled. Other exclusion criteria included known or suspected presence of any of the following conditions: clinically significant cardiovascular disease; severe dental disease; neurological disease, renal disease; liver disease (ALT levels of up to twice the upper reference limit and ALP levels of up to four times the upper reference limit were considered acceptable in the absence of other signs of liver disease); chronic pulmonary disease; infectious disease; immune-mediated disease; neoplasia; urinary tract infection; hypothyroidism (unless well controlled); diabetes mellitus; skin disease of the foot; obesity (nine out of the 1–9 body condition score scale). These were exclusion criteria because they may be associated with decreased activity that would not respond to analgesic treatment. Particular attention was given to ruling out neurological disease through a comprehensive neurological evaluation. Additionally, owners had to agree to not change the management of dogs for the period of the study, and owners were required to have a stable lifestyle for the duration of the study (no planned house moves, vacations, relationship changes or new pets).

### Inclusion Criteria for Part B

#### OA Group

Dogs were required to have clinical signs of pain in the sampled joint and radiographic evidence of OA in this joint, or osteoarthritic changes confirmed visually during opening of the joint (for euthanized cases). Dogs were not eligible for the study if immune-mediated disease was confirmed clinically, historically, and cytologically. Dogs were excluded from the study if no radiographic evidence of OA in the surgically treated joint. Cytology was performed on every sample by a cytopathologist (JA).

#### Control Group

Samples were obtained from elbows and stifles of dogs that were being euthanized for reasons unrelated to this study (population control). Inclusion in the control group required the absence of any signs of OA pain on examination prior to euthanasia, absence of joint OA on dissecting the joint post-mortem (no obvious cartilage damage and synovitis), and normal cytological evaluation of SF by a cytopathologist (JA).

### Study Protocol for Part A

The study was conducted over a 28-day period with outcome measures gathered at screening [Day-14 (D-14)] and on D0, D1, D14, and D28. The study protocol is outlined in Table 1. Approximately 14 days prior to starting the study, examinations, radiography of painful joints, and force plate gait analysis were performed to screen potential candidate dogs. Additionally, blood was drawn for complete blood count (CBC) and serum biochemistry. Urinalysis was also performed. Dogs that met our inclusion criteria were enrolled in the study and assigned a case number. An omni-directional activity monitor (Actical^®^: Respironics, Murrysville, PA, USA) was placed on the neck collar.

**Table 1:** Study outline.

**Day of study**	**Action**
Prior to veterinary hospital screening visit	• Screening of patients/owners over telephone • Review veterinary medical history
Screening (Day-14)	Screening of dog and owner: • Owner to complete informed consent • Physical, neurological & orthopedic examination • Radiographs of painful joints • CBC; chemistry panel; UA • Assignment of case number (if dog meets inclusion criteria) • Collect pre-study sample for PK and cytokine analyses • Collect FP data
Day 0	• Physical, neurological & orthopedic examination • Collect FP data *(baseline)*- define *index limb* • Collect blood samples for PK and cytokine (TNF-α) analyses prior to caTNFR-Fc fusion protein administration *(baseline)* • Collect synovial fluid sample prior to injection for cytokine analyses • Administer caTNFR-Fc fusion protein IA into index joint under sedation • Collect blood sample for PK and cytokine (TNF-α) analyses *at* ~*4 h post administration*
Day 1 (owner and dog to visit)	• Collect blood samples for PK and cytokine (TNF-α) analyses *at* ~*24 h post administration*
Day 14 (± 2 days)/week 2	• Physical, neurological & orthopedic examination • Collect FP data • Collect blood samples for PK and cytokine (TNF-α) analyses
Day 28 (± 2 days)/week 4	• Physical, neurological & orthopedic examination • Collect FP data • CBC; chemistry panel; UA • Collect blood samples for PK and cytokine (TNF-α) analyses • ELISA assay for neutralizing antibodies to caTNFR-Fc fusion protein

*CBC, complete blood count; UA, urinalysis; PK, pharmacokinetics; FP, force plate; IA, intra-articular*.

On D0, following the examinations and gait assessment, the dogs were given the IA injection of caTNFR-Fc protein under sedation (see below for the details). Following IA injection of caTNFR-Fc protein, the dogs were monitored for a period of 4 h for any signs of AEs. Blood was drawn before injection and 4 h after the injection for pharmacokinetics (PK) of caTNFR-Fc protein. All dogs re-visited the hospital 1 day after the injection for the purpose of evaluating for AEs and collecting serum samples for caTNFR-Fc protein PK. On D14 and D28 of the study, gait analysis, examinations, and blood collection for caTNFR-Fc protein PK were repeated. Additionally, blood and urine were collected at D28.

The owners were asked to report any changes following the injection (e.g., redness, pain, and swelling of the injection site).

### Sample Size Estimation

#### Part A

Sample size estimation was performed based on peak vertical force (PVF) data collected in dogs of a similar phenotype, who were receiving an NSAID. After 2 weeks of an NSAID, the average improvement ± SD in PVF was 4.39% body weight ±3.98. Assuming there would be no change from baseline with no treatment (a reasonable assumption for pilot data), and a significance level of 0.05, and a power of 0.8, a one-sample size estimation suggested nine dogs would be needed if the treatment produced the same effect as a systemic NSAID. Based on our experience, the screening failure rate of this particular study is 10%−20%, and total number of target enrolled dogs should be 1.2 times higher than the sample size estimation, so we targeted enrollment of 12 dogs.

#### Part B

Although one study measured TNF-α in SF samples from OA dogs using ELISA (12), TNF-α was not detected in any samples. Thus, to get a better idea of the sample size, sample size estimation was performed based on the previous study investigating TNF-α in SF in humans with chronic knee OA. Mean ± SD of TNF-α was 6.51 ± 2.41 in OA joints. Since there was no data from normal joints, we assumed that normal joints would have concentrations of 25% of that in OA joints. The difference between the groups is 4.88. Sample size was estimated with a significance level of 0.05, and a power of 0.8, and this suggested that a total of 12 dogs would be needed.

### Injection of the caTNFR-Fc Fusion Protein

On D0, following examination and gait assessment, each dog was sedated with dexmedetomidine (0.003 mg/kg) and hydromorphone (0.05 mg/kg) intravenously to facilitate IA administration of the caTNFR-Fc fusion protein; the affected joint was aseptically prepared. The injection was performed by the board-certified veterinarian (BDXL). Following the placement of a 22 G needle into the joint space, a SF sample (up to 1 ml) was obtained before IA injection to confirm the correct location of the needle (the SF contributed to samples analyzed in Part B). The needle was left in place while the syringe of SF was removed. A syringe containing 2.2 mg (1 ml) of the caTNFR-Fc fusion protein was attached and an injection was made. The dose of caTNFR-Fc fusion protein was extrapolated from the previous studies in humans based on the volume of SF and TNF-α in the OA knee joint (7) (see later for dose determination). Following injection, the joint was flexed and extended ~10 times to distribute the caTNFR-Fc fusion protein evenly within the joint space. After the IA injection of the caTNFR-Fc fusion protein, atipamezole (the same volume as dexmedetomidine) was administered intramuscularly to reverse the effects of dexmedetomidine, each dog was allowed to recover and was monitored for a period of 4 h for any signs of AEs, before blood was drawn for PK.

### Development of TNF Receptor Fc Fusion Protein

#### Canine TNF Receptor p80

The complete canine p80 TNFR amino acid sequence was assembled by combining the predicted carboxy-terminal sequence of NCBI genomic reference clone XP_544562.2 (containing an incorrectly predicted signal sequence) with the predicted signal sequence and amino-terminal sequence from the partial canine cDNA clone DN368636.

#### Construction

The p80 TNFR extracellular domain was attached N-terminally, *in silico*, to the Fc domain of canine IgG type B (13) and a gene was designed to express the fusion protein using codons selected for optimal expression in Chinese Hamster Ovary (CHO) cells. The gene was constructed (Catalent^®^, Madison, WI, USA) using overlapping oligonucleotides, cloned into a CHO cell expression vector, and transfected into CHO cells (caTNFR-Fc CHO). Following culture *in vitro*, media conditioned by the caTNFR-Fc CHO cells was harvested and the expression product purified using Protein A affinity capture chromatography (binding at pH 7 and elution at pH 5 to selectively purify dimeric caTNFR-Fc fusion protein from higher molecular weight aggregates). The caTNFR-Fc fusion protein was purified with a high yield (0.6 g/L). Analysis by SDS-PAGE showed the expected dimeric protein of ~100 kDa in non-reducing conditions and a monomer of 50 kDa in reducing conditions.

#### *In vitro* Efficacy Testing

The inhibition of canine TNF-α (R&D Systems^®^, 1 ng/ml) bioactivity by purified caTNFR-Fc was assessed using 293-HEK cells transfected with the NF-kB reporter construct pTRH1 (14). These cells respond to canine TNF-α by fluorescence. Canine TNF-induced fluorescence was completely inhibited by caTNFR-Fc fusion protein with an IC50 of ~1 ng/ml. In separate experiments, the caTNFR-Fc construct did not show detectable binding to complement C1q *in vitro*, suggesting that the caTNFR-Fc fusion protein would not initiate a complement cascade *in vivo*.

#### Dose Determination

Data from studies in humans indicate the mean SF volume of the knee is ~3.0–6.7 ml (15, 16), so we used 5.0 ml as a mean. A recent clinical trial showed that 10 mg of IA injection of Etanercept significantly improved OA-related clinical signs (7). Based on these results and our clinical estimation of the SF volume of an arthritic stifle or elbow in a dog to be ~1.0 ml, we aimed for 2.0 mg of the caTNFR-Fc fusion protein. Manufacturing considerations and volume of injectate considerations led to a final dose of 2.2 mg of the caTNFR-Fc fusion protein in a 1.0 ml volume.

#### *In vivo* Pharmacokinetics, Immunogenicity, and Safety

Following Institutional Ethics Panel review and approval, the purified caTNFR-Fc fusion protein was injected subcutaneously into 12 beagle dogs (Charles River Laboratories, Ballina, Ireland) and plasma samples were taken at various times following injection. The caTNFR-Fc fusion protein was detected in plasma by binding to canine TNF-α by enzyme-linked immunosorbent assay (ELISA) using secondary anti-canine IgG polyclonal antibody-HRP conjugate. The elimination half-life of the caTNFR-Fc fusion protein in dog plasma was determined in two dogs to be 3.5 and 3 days, respectively—a half-life equivalent to etanercept (4.3 days) *in vitro* and in safety test, respectively (6). Anti-caTNFR-Fc fusion protein antibodies were assessed using a competitive ELISA technique. Anti-TNFR-Fc protein antibodies were not detected in any of the 12 dogs tested following a single injection of 0.2 mg/kg caTNFR-Fc fusion protein. There were no AEs observed in any dogs injected with a caTNFR-Fc fusion protein, with a maximum dose tested of 0.5 mg/kg (*n* = 6).

### Outcome Measures for Part A

The primary outcome measure was the force plate gait analysis. The secondary outcome measures were owner-assessed side effects, hematology, clinical chemistry, and urinalysis.

#### Force Plate

Limb use was measured using dual in series force plates (AMTI, Watertown, MA, USA) and custom software (Sharon software, Dewitt, MI, USA). Velocity and acceleration were measured by means of five photoelectric cells placed 0.5 m apart and coupled with a triggered timer system. The dogs were trotted across the force plates at a comfortable speed. Velocity and acceleration of each dog was restricted to baseline values ± 0.2 and ± 0.5 m/s, respectively throughout the study period. A trial from which data was retained for analysis consisted of a full forefoot strike on each force plate without another foot being on the plate at the same time, followed by an ipsilateral hindfoot strike in the same fashion on each force plate. Thus, data from all four limbs were obtained in a single pass. A single trained observer (ME) evaluated each foot strike and subsequent force profile and determined whether or not the trial should be retained. A single handler (BC) gaited all the dogs for each trial and timepoint. Five valid trials were collected for each dog at each timepoint. PVF and vertical impulse (VI) were the GRFs extracted, and the means of the five trials at each visit were used for analysis. All forces were normalized to percent body weight. The change from baseline (D0) in PVF and VI were calculated for analysis. Symmetry Index (SI) for PVF and VI were calculated using the following formula (17):


$$SI=\left(X_{i}-X_{j}\right)/0.5\left(X_{i}+X_{j}\right)\times 100\;$$

where *X*_i_ is the mean of the index limb and *X*_j_ is the mean of the non-index limb. An SI of 0 means there is perfect symmetry between the forelimbs or hindlimbs, and a value of −200 means the dog is non-weight bearing on the index limb.

#### Accelerometry

As previously reported, dogs were fitted with a collar-mounted accelerometer throughout the study period (D-14 to D28) to continuously record activity (18). Activity count over 1 week prior to each outcome measure time point (D0, D14 and D28) has been calculated, and expressed as mean activity count per minute over each 7-day period. Owners were asked to keep a diary of any unusual events that might affect a dog's activity. The change from baseline in activity count was also calculated for analysis.

#### Clinical Metrology Instruments

Mobility impairment was assessed through a review of LOAD, which was completed by the same owner at all visits. The change from baseline in LOAD was calculated for analysis.

#### Pharmacokinetics

The concentration of circulating caTNFR-Fc fusion protein was measured as described above. The lower limit of quantitation was 500 pg/ml. Plasma samples were stored at −80°C until use.

#### ELISA for Neutralizing Antibodies to the caTNFR-Fc Fusion Protein

Plasma samples were assayed using a competitive ELISA for inhibition of binding of the purified caTNFR-Fc fusion protein to mouse TNF. The purified caTNFR-Fc fusion protein was mixed with dog plasma from caTNFR-Fc fusion protein treated dogs and added to plates previously coated with mouse TNF. Following incubation, blocking and washing, binding was detected using secondary anti-canine IgG polyclonal antibody-HRP conjugate. Purified caTNFR-Fc fusion protein without dog plasma, in the absence or presence of a neutralizing anti-caTNFR-Fc fusion protein mouse monoclonal antibody (1RC1), was used as negative and positive controls.

### Outcome Measures for Part B

#### ELISA for SF and Serum

SF samples were centrifuged at 4°C, 3,000 × g, for 20 min and the serum samples were centrifuged at 4°C, 2,000 × g, for 10 minutes. Within 2 h of collection, supernatants of SF and serum were stored at −80°C for analysis until use. A commercially available ELISA kit (Sigma-Aldrich^®^, Saint Louis, MO, USA) was used to measure the concentration of TNF-α in samples, in triplicate, without dilution, according to the manufacturer's protocols. The detection limit of the assay was 2 pg/ml.

### Statistical Analysis

Statistical analyses were performed using JMP^®^ software (JMP^®^ Pro 13, SAS^®^, Cary, NC, USA). In all analyses, the critical *p*-value was set as 0.05.

#### Part A

A one-way analysis of variance (ANOVA) was used to evaluate the overall effect of time on GRFs. LOAD, and accelerometry data.

#### Part B

TNF-α concentrations were compared between groups using Wilcoxon's signed-rank test.

## Results

### Part A

The study was conducted between January and May 2016. Seventy-one inquiries from interested dog owners were received and following a telephone discussion, this resulted in 29 screening appointments. Of these, there were 17 screening failures: seven for insufficient or inconsistent lameness; three for lameness due to coxofemoral joint pain; three for being unable to perform force plate gait analysis; one for lack of adequate joint pain response; one for neurological disease; one for suspected joint infection; one for concurrent exclusionary disease. One dog was withdrawn from the study several days after injection due to a family problem. Baseline characteristics of the dogs enrolled in the study (and used for evaluation of efficacy) are summarized in Table 2. Breeds included were three Labrador retrievers, three mixed-breed dogs, two Catahoula leopard dogs, and one each of Great Pyrenees, German shepherd, American Staffordshire terrier, and Australian cattle dog. A single joint was injected in each dog—seven elbows, four stifles, and one tarsal joint.

**Table 2:** Characteristics of each dog enrolled into Part A study.

**Dog ID**	**Sex**	**Age (year)**	**Breed**	**Weight (kg)**	**Index joint**	**LOAD Score at screening (Day−14)**
1	MC	3.9	Great pyrenes	45.2	R stifle	18
2	FS	7.2	Catahoula leopard dog	36.7	R elbow	26
3	M	1.2	German shepherd dog	41.8	R elbow	14
4	MC	6	Labrador retriever	35.4	L elbow	16
5	MC	5	American Staffordshire	35.8	L elbow	21
6 #	MC	4.1	Australian cattle dog	27.9	L stifle	36
7	FS	11.1	Labrador retriever	35.3	R tarsus	27
8	MC	12.4	Mixed breed	32.6	R elbow	24
9	MC	9.2	Mixed breed	27.1	R stifle	20
10	FS	12.7	Labrador retriever	42.9	R stifle	31
11	FS	7.3	Catahoula leopard dog	31.2	L elbow	35
12	MC	12.2	Mixed breed	30.2	L elbow	23

*FS, female spayed; MC, male castrated; M, male; L, left; R, right; LOAD, Liverpool Osteoarthritis in dogs*.

*^#^This dog excluded from analysis*.

#### Laboratory Data

Occasional values were not within the reference intervals; none of them were clinically significant at either the start or end of the study (data not shown).

#### Force Plate

Mean ± SD of PVF was 75.8 ± 18.9, 75.0 ± 18.0, and 75.7 ± 18.8, and VI was 12.3 ± 3.5, 12.1 ± 3.3, and 12.4 ± 3.6 at D0, D14, and D28, respectively. Overall, there was no effect of time on GRFs (*p* = 0.99 for PVF and 0.90 for VI; Tables 3a,b).

**Table 3a:** Outcome measure values at each time point in Part A of the study (mean ± SD and range).

**Outcome measures**	**Day 0**	**Day 14**	**Day 28**	**Overall time effect (*p*-value)**
LOAD (0 to 52)	23.8 ± 5.1 (17 to 33)	19.2 ± 3.8 (14 to 36)	19.5 ± 5.9 (10 to 34)	0.057
PVF (%BW)	75.8 ± 18.9 (49.5 to 104.8)	75.0 ± 18.0 (50.8 to 103.6)	75.7 ± 18.8 (52.4 to 109.2)	0.99
VI (%BW·S)	12.3 ± 3.5 (6.8 to 16.9)	12.1 ± 3.3 (7.0 to 16.0)	12.4 ± 3.6 (7.0 to 18.2)	0.90
SI for PVF	−19.5 ± 10.2 (−40.3 to −7.3)	−20.3 ± 10.0 (−38.1 to −5.0)	−16.8± 8.9 (−38.9 to −7.3)	0.52
SI for VI	−16.4 ± 9.8 (−35.7 to −5.2)	−15.9 ± 10.5 (−33.7 to −3.3)	−14.1 ± 7.1 (−32.2 to −6.2)	0.55
Accelerometry	64.7 ± 35.3 (38.3 to 163.2)	67.7 ± 32.0 (36.6 to 143.9)	73.9 ± 46.1 (32.8 to 207.3)	0.62

*All kinetic variables (PVF and VI) normalized to percent body weight*.

*LOAD, Liverpool Osteoarthritis in Dogs; PVF, peak vertical force; VI, vertical impulse; SI, symmetry index*.

*Statistically significant: p < 0.05*.

**Table 3b:** Changes from baseline in outcome measures at each time point in Part A of the study study (mean ± SD and range).

**Outcome measures**	**Day 14**	**Day 28**
LOAD (0 to 52)	−4.6 ± 3.5 (−11 to 0)	−4.3 ± 5.2 (−15 to −1)
PVF (%BW)	−0.8 ± 3.2 (−5.9 to 4.1)	−0.1 ± 5.9 (−11.9 to 9.9)
VI (%BW·S)	−0.2 ± 0.4 (−0.9 to 0.4)	0.2 ± 1.0 (−1.7 to 2.0)
SI for PVF	−0.8 ± 4.0 (−6.4 to 7.1)	2.1 ± 9.7 (−15.0 to 19.6)
SI for VI	0.6 ± 3.5 (−4.0 to 5.7)	−1.9 ± 8.7 (−12.4 to 21.5)
Accelerometry	2.9 ± 16.0 (−19.3 to 32.6)	9.1 ± 14.7 (−8.2 to 44.0)

*All kinetic variables (PVF and VI) normalized to percent body weight*.

*LOAD, liverpool osteoarthritis in Dogs; PVF, peak vertical force; VI, vertical impulse; SI, symmetry index*.

**Figure 1:**
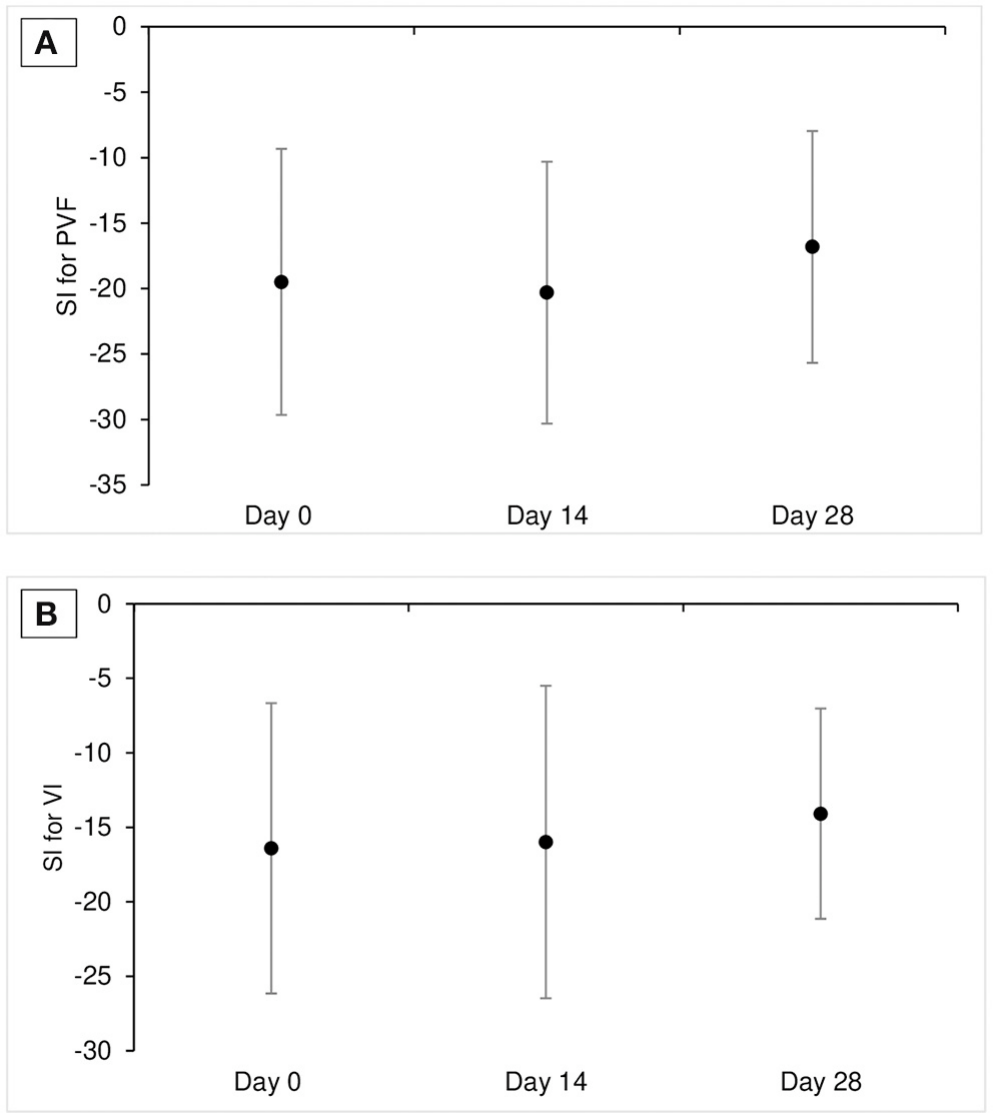
Mean ± SD SI for PVF **(A)** and SI for VI **(B)** at each time point in Part A study (*n* = 11). SI value of 0 means perfect symmetry between affected limb pairs and negative SI value means that the dogs put less weight on the affected limb. SI, symmetrical index; PVF, peak vertical force; VI, vertical impulse.

Mean ± SD of SI for PVF was −19.5 ± 10.2, −20.3 ± 10.0, and −16.8± 8.9, and mean ± SD of SI for VI was −16.4 ± 9.8, −15.9 ± 10.5, and −14.1 ± 7.1 at D0, D14, and D28, respectively. There was no significant change over time in SI values for PVF for VI (*p* = 0.52 for SI for PVF and 0.55 for SI for VI; Tables 3a,b, and Figure 1).

#### LOAD

**Figure 2:**
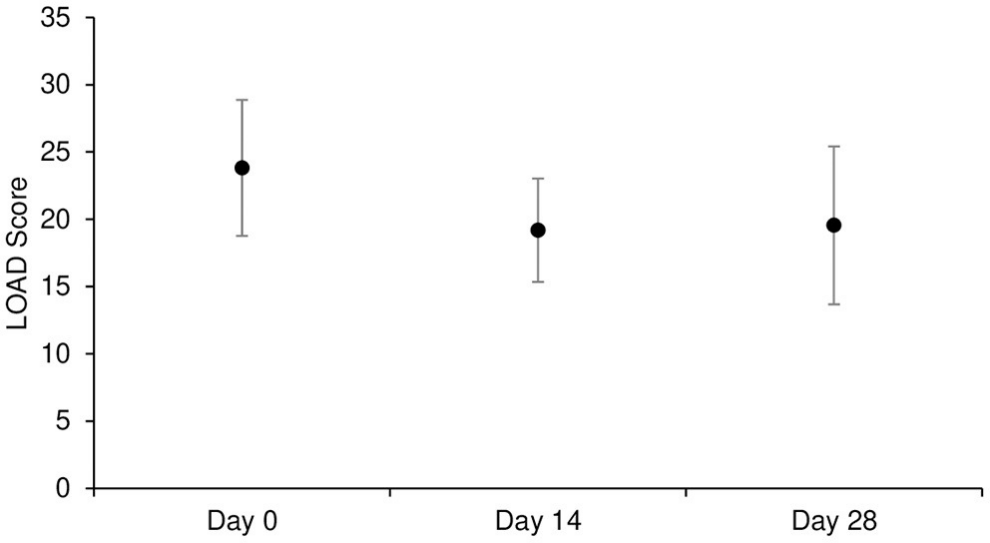
Mean ± SD of LOAD score at each time point in Part A study (*n* = 11). LOAD: Liverpool Osteoarthritis in Dogs.

Mean ± SD of LOAD was 23.8 ± 5.1, 19.2 ± 3.8, 19.5 ± 5.9 at D0, D14, and D28, respectively. Overall, there was no significant time effect on LOAD score (*p* = 0.057; Tables 3a,b, and Figure 2).

#### Accelerometry

Mean ± SD of mean activity count per minute over the preceding week was 64.7 ± 35.3, 67.7 ± 32.0, and 73.9 ± 46.1 at D0, D14, and D28, respectively. Overall, there was no effect of time on mean hourly activity counts (*p* = 0.62; Tables 3a,b).

#### Pharmacokinetics

caTNFR-Fc fusion protein could not be detected in plasma at any time points in any dogs.

#### Owner-assessed Side Effects

No side effects were reported.

### Part B

#### Synovial fluid

**Figure 3:**
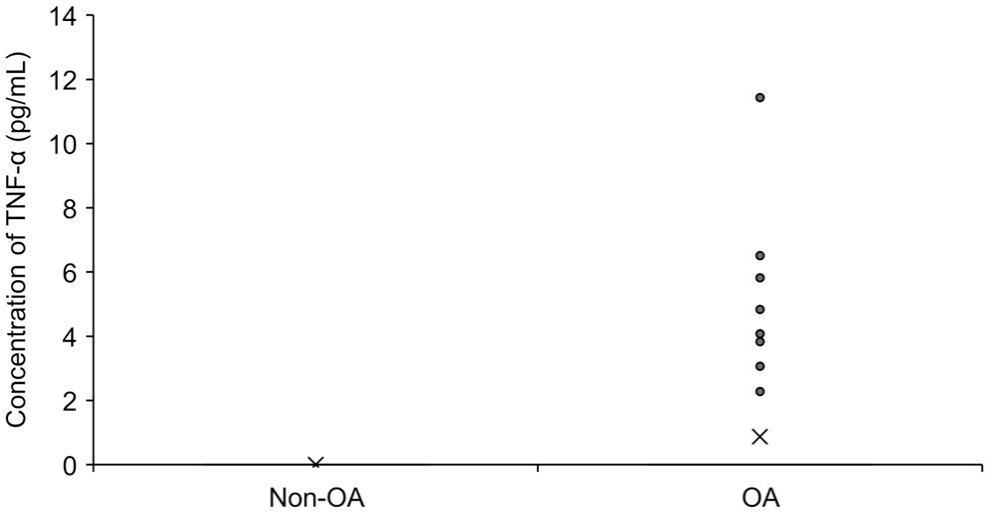
The concentration of TNF-α in synovial fluid in dogs with OA (*n* = 69) and without OA (*n* = 79). The mean value is denoted as ‘x'. OA: osteoarthritis.

A total of 69 SF samples (57 dogs) from OA joints and 79 samples (39 dogs) from normal joints were analyzed. The dogs who received surgery had a history of lameness duration of 0.25 months to 48 months (median of 5 months) and all dogs had radiographic signs of OA in the treated joints. The concentration of TNF-α was significantly higher in OA joints than in healthy joints (*p* = 0.0019). However, TNF-α was detected only in 10 out of 69 SF samples from OA joints (14%) and was not detected in any of the normal joints (Figure 3). Sufficient SF for analysis was collected from nine dogs that participated in Part A and TNF-α was only detected in two samples. Twenty-seven OA dogs (29 joints) had received NSAIDs, three OA dogs (four joints) had received medication other than NSAIDs (tramadol, gabapentin, antibiotics), five OA dogs (five joints) had received supplement (fish-oil and glucosamine), and 22 OA dogs (31 joints) had not received any medication prior to the sample collection. No obvious abnormalities were observed in cytology in any SF samples.

#### Serum

**Figure 4:**
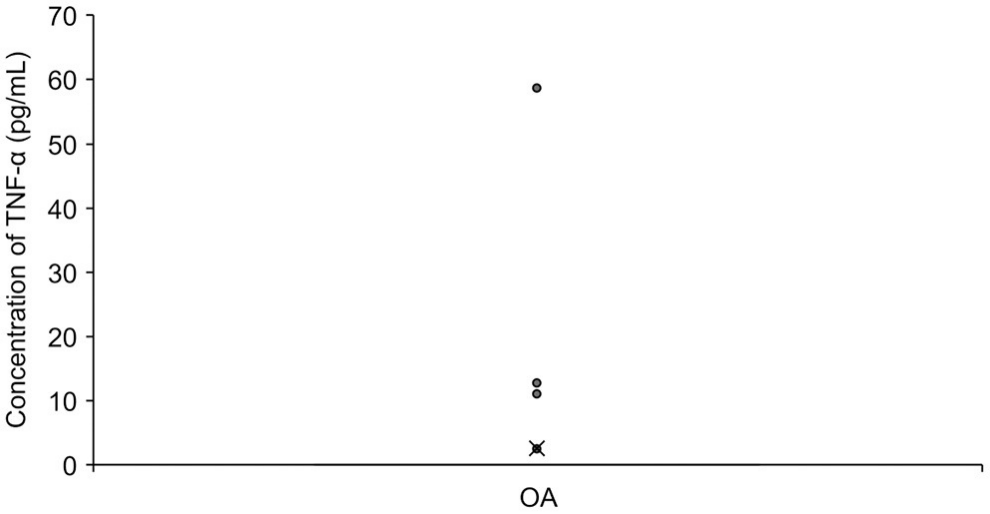
The concentration of TNF-α in Serum in OA dogs (*n* = 50). The mean value is denoted as ‘x'. OA: osteoarthritis.

A total of 50 serum samples from OA dogs were analyzed, but TNF-α was detected in only three of them (two dogs in the Part A study). Twenty-five OA dogs had received NSAIDs prior to the sample collection (Figure 4).

TNF-α was detectable in two dogs in both SF and serum. The intra-assay coefficient of variability for ELISA was <10% in both serum and SF samples.

## Discussion

In this study, we evaluated the efficacy of IA injection of a TNF receptor Fc fusion protein for the management of OA-pain in dogs using objective gait analysis and objective accelerometry, and also subjective owner assessments. The results showed that an IA injection of the fully caTNFR-Fc fusion protein provided no detectable benefit in limb use over the 4-week time period in dogs with OA-pain. In additional work, we found that TNF-α concentrations in SF was significantly higher in OA joints compared to healthy joints, but the detection of TNF-α in synovial fluid was infrequent. An IA injection of the caTNFR-Fc fusion protein may not be effective for the management of OA-associated pain in dogs, and TNF-α may not be an important mediator in canine OA.

TNF-α has been described as playing a role in inflammation and pain in OA, as well as a role in cartilage degradation (3). However, despite this, it is not known what the therapeutic effect (clinical benefit in OA patients) of targeting TNF-α is. To the best of our knowledge, only two open-label studies have been published which evaluate the efficacy of IA injection of anti-TNF therapy (adalimumab and etanercept) compared with hyaluronic acid (HA) and assessed outcome using PROs (7, 19). Although these studies concluded that a single injection of anti-TNF therapy was superior to HA over the 4-week time period, such open-label studies using subjective PROs are subject to potential placebo effects. Indeed, the placebo effect in studies of IA therapeutics is particularly strong (8). Although our study was “open-label,” and not placebo-controlled, we employed objective gait analysis to evaluate the outcome. Gait analysis and activity monitors have been validated as a surrogate of joint pain in dogs by virtue of the fact that positive changes are seen in dogs with joint pain who receive known analgesics (20, 21). Our results showed that the caTNFR-Fc fusion protein failed to improve limb use following injection IA. Although we used objective measures of limb use, a placebo-controlled study, even with objective measures, would need to be employed before definitive statements on efficacy can be made. The apparent lack of efficacy in limb use may be explained by the short half-life of the caTNFR-Fc fusion protein and the low and inconsistently measurable concentrations of TNF-α in the OA joint. The half-life time of caTNFR-Fc fusion protein utilized is equivalent to that of etanercept (~3.5 days) in healthy dogs. However, the hinge region of anti-TNF-α agents is known to be recognized by proteases such as matrix metalloproteinase (MMP), which is significantly elevated in SF in canine OA joints compared to normal joints (22). Previous *in vitro* work has shown that metalloproteinases can cleave etanercept into an Fc domain and an extracellular domain of TNF receptor (23), and the soluble TNF-receptor isolated from etanercept was not able to neutralize TNF (23). Thus, the half-life of caTNFR-Fc fusion protein in OA joints may be much shorter than in the systemic circulation, and any effect of caTNFR-Fc fusion protein on pain might have dissipated by the time of outcome measure collections. Future work should evaluate the half-life of caTNFR-Fc fusion protein in SF from arthritic joints and consider repeated injections. In this study, caTNFR-Fc fusion protein was injected into various joints with varying stages of disease and pain. Previous studies in humans showed that cytokine levels, including TNF-a, may be influenced by various factors, such as age (young vs. geriatric), joint affected, and stage of OA (acute vs. chronic) (24–26). Additionally, ideally, the quantity of caTNFR-Fc fusion protein injected, and the volume, should have been adjusted based on joint size. However, in this initial proof of concept study, we considered our approach to total dose and volume to be an appropriate starting point. Future assessments of efficacy could incorporate sufficient data to control for these factors.

Previous studies have measured TNF-α concentrations in both healthy joint SF, and SF from OA joints. Several studies found that the concentration of TNF-α in SF was significantly higher in joints with OA secondary to hip dysplasia or cruciate ligament rupture compared to healthy joints (22, 27). However, one study showed that TNF-α activity was significantly lower in the SF in OA joints secondary to cruciate ligament rupture compared to healthy joints, and another study demonstrated that there was no significant difference in mRNA expression of TNF-α between healthy stifle joints and OA stifle joints (28, 29). In the present study, there was a significant difference in TNF-α concentration in SF between the OA joints (higher concentrations) and the normal joints, however, the detection of TNF-α in SF samples was infrequent. Although the infrequent detection is consistent with previous work (12), this also reflects a limitation of our study. The infrequent detection may be explained by the lower sensitivity of the ELISA kit or the use of NSAIDs in some dogs. First, the sensitivity of the ELISA kit employed in this study was 2 pg/ml. Although this is the lowest limit of quantification among commercially available canine TNF ELISA kits as far as we know, this may not be sensitive enough based on the published study in humans, which found that the mean TNF-α concentration in SF with late stage of OA was 0.124 pg/ml (30). Second, it has been reported that NSAIDs may decrease concentrations of TNF-α in SF in humans (31). As some surgery cases had received NSAIDs before surgery, TNF-α concentration might be decreased by NSAIDs. Further study is needed to conclude if TNF-α can be elevated in OA joints. Other potential factors that may have influenced our results are the varying causes of OA in joints sampled (cruciate rupture, fragmented coronoid process), varying age of dogs, and stage or severity of OA. Future work could aim to control for these factors to further explore whether TNF-α is an important mediator in canine OA and associated pain. Previous work has suggested that the concentration of TNF-α in SF may vary depending on the level of inflammation and stage of OA (22). Future work should investigate this further, as this may lead to a rationale for using anti-TNF-α therapy in certain dogs with certain stages of joint inflammation and OA (22, 27).

TNF-α was infrequently detected in serum in dogs with OA in our study. Our data reflects the results of a recent study where TNF-α was measured in the serum of OA dogs using a multiplex assay– that study showed that the majority of OA dogs had <2.0 pg/ml of TNF-α (32) in their blood.

One of the major concerns of blocking the actions of TNF-α, although the risk is small, is the increased risk of serious opportunistic infection due to its immunosuppressant effect (33). Generally, local administration could reduce systemic exposure, which leads to fewer off-target effects and AEs. However, rapid systemic absorption of TNF-α blockers *via* inflamed synovium following IA injection still remains a concern (34). Indeed, systemic effects after IA injection of the anti-TNF-α have been reported (35). The concentration of circulating caTNFR-Fc fusion protein following IA injection was measured in our study and showed that caTNFR-Fc fusion protein either did not diffuse out of the joint rapidly following IA administration, or was below the limit of quantification in serum. Additionally, systemic AEs were not observed in the present study.

This non-randomized, open-label, the pilot study indicated IA injection of caTNFR-Fc fusion protein did not improve limb function in dogs. Objective measured limb use measurement did not show significant improvement over time. Furthermore, although the concentration of TNF-α was significantly higher in SF from OA joints compared to that from healthy joints, very few dogs with OA had measurable SF concentrations of TNF-α.

## Data Availability Statement

The raw data supporting the conclusions of this article will be made available by the authors, without undue reservation.

## Ethics Statement

The animal study was reviewed and approved by North Carolina State University, The Institutional Animal Care and Use Committee. Written informed consent was obtained from the owners for the participation of their animals in this study.

## Author Contributions

BDXL and DG: study design. BC and ME: acquisition of data. BDXL, ME, and JA: analysis and interpretation of data. ME, AN, DG, and BDXL: manuscript preparation and statistical analysis. All authors contributed to the article and approved the submitted version.

## Funding

The study was funded by Nexvet (internal grant number: 2016-1534).

## Conflict of Interest

BDXL was a paid consultant for Nexvet and DG was a paid employee of Nexvet. The remaining authors declare that the research was conducted in the absence of any commercial or financial relationships that could be construed as a potential conflict of interest.

## Publisher's Note

All claims expressed in this article are solely those of the authors and do not necessarily represent those of their affiliated organizations, or those of the publisher, the editors and the reviewers. Any product that may be evaluated in this article, or claim that may be made by its manufacturer, is not guaranteed or endorsed by the publisher.

## References

[1] Johnston SA. Osteoarthritis. Joint anatomy, physiology, and pathobiology. Vet Clin North Am Small Anim Pract. (1997) 27:699–723. 10.1016/S0195-5616(97)50076-3 9243777

[2] Wright AK Amodie D Cernicchiaro N Lascelles BDX Pavlock A. In-clinic functional tests in dogs with osteoarthritis show progressive improvement with prolonged carprofen administration: results of a pilot study of 133 dogs. J Small Anim Pract. (2022) accepted. 10.1111/jsap.13500 35385129 PMC9543207

[3] Kalliolias GD Ivashkiv LB. TNF biology, pathogenic mechanisms and emerging therapeutic strategies. Nat Rev Rheumatol. (2016) 12:49–62. 10.1038/nrrheum.2015.169 26656660 PMC4809675

[4] Schaible HG. Nociceptive neurons detect cytokines in arthritis. Arthritis Res Ther. (2014) 16:470. 10.1186/s13075-014-0470-8 25606597 PMC4289196

[5] Mitoma H Horiuchi T Tsukamoto H Ueda N. Molecular mechanisms of action of anti-TNF-alpha agents - comparison among therapeutic TNF-alpha antagonists. Cytokine. (2018) 101:56–63. 10.1016/j.cyto.2016.08.014 27567553

[6] Scott LJ. Etanercept: a review of its use in autoimmune inflammatory diseases. Drugs. (2014) 74:1379–410. 10.1007/s40265-014-0258-9 25034360

[7] Ohtori S Orita S Yamauchi K Eguchi Y Ochiai N Kishida SK. Efficacy of direct injection of etanercept into knee joints for pain in moderate and severe knee osteoarthritis. Yonsei Med J. (2015) 56:1379–83. 10.3349/ymj.2015.56.5.1379 26256983 PMC4541670

[8] Previtali D Merli G Frattura GD Candrian C Zaffagnini S Filardo G. The long-lasting effects of “placebo injections” in knee osteoarthritis: a meta-analysis. Cartilage. (2020) 13(1_suppl):185S−96S. 10.1177/1947603520906597 32186401 PMC8808779

[9] Philp AM Davis ET Jone SW. Developing anti-inflammatory therapeutics for patients with osteoarthritis. Rheumatology. (2017) 56:869–81. 10.1093/rheumatology/kew278 27498352

[10] Walton MB Cowderoy E Lascelles D Innes JF. Evaluation of construct and criterion validity for the 'Liverpool Osteoarthritis in Dogs' (LOAD) clinical metrology instrument and comparison to two other instruments. PLoS ONE. (2013) 8:e58125. 10.1371/journal.pone.0058125 23505459 PMC3591443

[11] Hercock CA Pinchbeck G Giejda A Clegg PD Innes JF. Validation of a client-based clinical metrology instrument for the evaluation of canine elbow osteoarthritis. J Small Anim Pract. (2009) 50:266–71. 10.1111/j.1748-5827.2009.00765.x 19527419

[12] Little JP Bleedorn JA Sutherland BJ Sullivan R Kalscheur VL Ramaker MA. Arthroscopic assessment of stifle synovitis in dogs with cranial cruciate ligament rupture. PLoS ONE. (2014) 9:e97329. 10.1371/journal.pone.0097329 24892866 PMC4043664

[13] Tang L Sampson C Dreitz MJ McCall C. Cloning and characterization of cDNAs encoding four different canine immunoglobulin gamma chains. Vet Immunol Immunopathol. (2001) 80:259–70. 10.1016/S0165-2427(01)00318-X 11457479

[14] Vince JE Wong WW Khan N Feltham R Chau D Ahmed AU. IAP antagonists target cIAP1 to induce TNF alpha-dependent apoptosis. Cell. (2007) 131:682–93. 10.1016/j.cell.2007.10.037 18022363

[15] Kraus VB Stabler TV Kong SY Varju G McDaniel G. Measurement of synovial fluid volume using urea. Osteoarthritis Cartilage. (2007) 15:1217–20. 10.1016/j.joca.2007.03.017 17507255 PMC2034527

[16] Heilmann HH Lindenhayn K Walther HU. Synovial volume of healthy and arthrotic human knee joints. Z Orthop Ihre Grenzgeb. (1996) 134:144–8. 10.1055/s-2008-1039786 8779258

[17] Volstad NJ Sandberg G Robb S Budsberg SC. The evaluation of limb symmetry indices using ground reaction forces collected with one or two force plates in healthy dogs. Vet Comp Orthopaed. (2017) 30:54–8. 10.3415/VCOT-16-04-0054 27849103

[18] Hansen BD Lascelles DX Keene BW Adams AK Thomson AE. Evaluation of an accelerometer for at-home monitoring of spontaneous activity in dogs. Am J Vet Res. (2007) 68:468–75. 10.2460/ajvr.68.5.468 17472445

[19] Wang JP. Efficacy and safety of adalimumab by intra-articular injection for moderate to severe knee osteoarthritis: an open-label randomized controlled trial. J Int Med Res. (2018) 46:326–34. 10.1177/0300060517723182 28840750 PMC6011328

[20] Brown DC Boston RC Farrar JT. Comparison of force plate gait analysis and owner assessment of pain using the Canine Brief Pain Inventory in dogs with osteoarthritis. J Vet Intern Med. (2013) 27:22–30. 10.1111/jvim.12004 23311715

[21] Brown DC Boston RC Farrar JT. Use of an activity monitor to detect response to treatment in dogs with osteoarthritis. J Am Vet Med Assoc. (2010) 237:66–70. 10.2460/javma.237.1.66 20590496 PMC2905214

[22] Fujita Y Hara Y Nezu Y Yamaguchi S Schulz KS Tagawa M. Direct and indirect markers of cartilage metabolism in synovial fluid obtained from dogs with hip dysplasia and correlation with clinical and radiographic variables. Am J Vet Res. (2005) 66:2028–33. 10.2460/ajvr.2005.66.2028 16379642

[23] Biancheri P Brezski RJ Di Sabatino A Greenplate AR Soring KL Corazza GR. Proteolytic cleavage and loss of function of biologic agents that neutralize tumor necrosis factor in the mucosa of patients with inflammatory bowel disease. Gastroenterology. (2015) 149:1564–74. 10.1053/j.gastro.2015.07.002 26170138

[24] Bigoni M Sacerdote P Turati M Franchi S Gandolla M Gaddi D. Acute and late changes in intraarticular cytokine levels following anterior cruciate ligament injury. J Orthop Res. (2013) 31:315–21. 10.1002/jor.22208 22886741

[25] Ren G Lutz I Railton P Wiley JP McAllister J Powell J Krawetz RJ. Serum and synovial fluid cytokine profiling in hip osteoarthritis: distinct from knee osteoarthritis and correlated with pain. BMC Musculoskelet Disord. (2018) 5:19.39. 10.1186/s12891-018-1955-4 29402254 PMC5800026

[26] Michaud M Balardy L Moulis G Gaudin C Peyrot C Vellas B. Proinflammatory cytokines, aging, and age-related diseases. J Am Med Dir Assoc. (2013) 14:877–82. 10.1016/j.jamda.2013.05.009 23792036

[27] Fujita Y Hara Y Nezu Y Schulz KS Tagawa M. Proinflammatory cytokine activities, matrix metalloproteinase-3 activity, and sulfated glycosaminoglycan content in synovial fluid of dogs with naturally acquired cranial cruciate ligament rupture. Vet Surg. (2006) 35:369–76. 10.1111/j.1532-950X.2006.00159.x 16756618

[28] de Bruin T de Rooster H van Bree H Duchateau L Cox E. Cytokine mRNA expression in synovial fluid of affected and contralateral stifle joints and the left shoulder joint in dogs with unilateral disease of the stifle joint. Am J Vet Res. (2007) 68:953–61. 10.2460/ajvr.68.9.953 17764409

[29] Hay CW Chu QL Budsberg SC Clayton MK Johnson KA. Synovial fluid interleukin 6, tumor necrosis factor, and nitric oxide values in dogs with osteoarthritis secondary to cranial cruciate ligament rupture. Am J Vet Res. (1997) 58:1027–32. 9285010

[30] Özler K Aktaş E Atay Ç Yilmaz B Arikan M Güngör S. Serum and knee synovial fluid matrix metalloproteinase-13 and tumor necrosis factor-alpha levels in patients with late-stage osteoarthritis. Acta Orthop Traumatol Turc. (2016) 50:356–61. 10.3944/AOTT.2015.15.0115 27130394

[31] Gallelli L Galasso O Falcone D Southworth S Greco M Ventura V. The effects of nonsteroidal anti-inflammatory drugs on clinical outcomes, synovial fluid cytokine concentration and signal transduction pathways in knee osteoarthritis A randomized open label trial. Osteoarthritis Cartilage. (2013) 21:1400–8. 10.1016/j.joca.2013.06.026 23973155

[32] Muller C Enomoto M Buono A Steiner JM Lascelles BDX. Placebo-controlled pilot study of the effects of an eggshell membrane-based supplement on mobility and serum biomarkers in dogs with osteoarthritis. Vet J. (2019) 253:105379. 10.1016/j.tvjl.2019.105379 31685140

[33] Ali T Kaitha S Mahmood S Ftesi A Stone J Bronze MS. Clinical use of anti-TNF therapy and increased risk of infections. Drug Healthc Patient. (2013) 5:79–99. 10.2147/DHPS.S28801 23569399 PMC3615849

[34] Haroon M O'Gradaigh D. Efficacy and safety of combining intra-articular methylprednisolone and anti-TNF agent to achieve prolonged remission in patients with recurrent inflammatory monoarthritis. Joint Bone Spine. (2010) 77:232–4. 10.1016/j.jbspin.2010.02.008 20363657

[35] Bello S Bonali C Serafino L Rotondo C Terlizzi N Lapadula G. Intra-articular therapy with tumor necrosis factor-alpha antagonists: an update. Reumatismo. (2013) 65:257–63. 10.4081/reumatismo.2013.721 24705028

